# Low depression rates among caregivers of young children with sickle cell disease: a rapid report

**DOI:** 10.1093/jscdis/yoag014

**Published:** 2026-02-27

**Authors:** Catherine R Hoyt, Yuri Kim, Sophia C Larson, Hunter G Moore, Erin Macarthur, Allison A King, Andrew M Heitzer

**Affiliations:** Program in Occupational Therapy, Washington University School of Medicine, St. Louis, MO, 63110, United States; Department of Pediatrics, Washington University School of Medicine, St. Louis, MO, 63110, United States; Department of Neurology, Washington University School of Medicine, St. Louis, MO, 63110, United States; Cornell University, Ithaca, NY, 14853, United States; Program in Occupational Therapy, Washington University School of Medicine, St. Louis, MO, 63110, United States; Program in Occupational Therapy, Washington University School of Medicine, St. Louis, MO, 63110, United States; Department of Psychology & Biobehavioral Sciences, St Jude Children’s Research Hospital, Memphis, TN, 38105, United States; Program in Occupational Therapy, Washington University School of Medicine, St. Louis, MO, 63110, United States; Department of Pediatrics, Washington University School of Medicine, St. Louis, MO, 63110, United States; Department of Medicine, Washington University School of Medicine, St. Louis, MO, 63110, United States; Department of Surgery, Washington University School of Medicine, St. Louis, MO, 63110, United States; Department of Education, Washington University in St. Louis, St. Louis, MO, 63110, United States; Department of Psychology & Biobehavioral Sciences, St Jude Children’s Research Hospital, Memphis, TN, 38105, United States

**Keywords:** sickle cell disease, caregiver, mental health

## Abstract

Sickle cell disease (SCD) can bring lifelong challenges; new research reveals that caregivers of very young children with SCD do not experience high levels of depression on a short screening tool called the PROMIS.

In a study of 47 caregivers of children 0-5 years old with SCD, 94% responded similarly to the general population, with 3 (6%) caregivers reporting elevated depression.

These findings suggest a potential window of opportunity to connect caregivers of children with SCD to early interventions and family support. These early years may represent an optimal time for preventive interventions. Clinicians could refer to and implement family-centered support programs in the first years of life, rather than waiting and responding if crises emerge and disease complications may intensify.

The findings suggest that preventive care could leverage existing family strengths during these early years. This approach could inform how we support families navigating pediatric chronic illness, potentially altering long-term outcomes for both children and their caregivers.

## INTRODUCTION

Sickle cell disease (SCD) presents with complications that can emerge in the first years of life, including severe pain, anemia, and neurocognitive delays, particularly affecting cognitive and language development.^1^^,^^2^ Managing symptoms of SCD can be difficult for caregivers because of limited access to specialized SCD providers, which can result in extended wait times. When families must rely on providers unfamiliar with SCD, they may encounter biases such as pain crises being dismissed as drug-seeking behavior or underestimation of disease severity.^3^^,^^4^ Families with young children with SCD navigate a healthcare system overwhelmed by structural barriers that can make managing their child’s healthcare difficult and can cause psychological distress.^4^^,^^5^ Caregivers of children with other chronic conditions have reported increased symptoms of depression,^6^ yet studies of caregivers of children with SCD show mixed results, with some finding elevated depression while others report rates similar to the general population.^7^^,^^8^ Studies have also documented elevated caregiver stress in pediatric SCD related to disease management, hospitalizations, and chronic pain, though the association of stress to SCD-related factors varies across studies.^9–11^ Caregiver depression can affect treatment adherence, healthcare utilization, and child developmental outcomes, making early identification important for optimizing both family functioning and disease management. Environmental factors may compound these challenges, as families affected by SCD disproportionately live in under-resourced communities with limited access to healthcare and social supports, potentially amplifying caregiver psychological burden.^12^^,^^13^ However, many complications associated with SCD, such as cognitive deficits and pain, can intensify over time and may influence the timing of when caregiver mental health concerns emerge.

Caregivers of young children with SCD may not have experienced the most significant disease-related challenges (eg, severe pain, stroke). Prior studies examining caregiver depression in SCD have focused on families with older children, using instruments that are time-consuming and/or costly, preventing widespread implementation of caregiver mental health screening in SCD clinical care.^14–17^ Without broader screening, the understanding of when caregivers of children with SCD need the most support is limited.^7^ Thus, it remains unclear if many caregivers experience clinically significant levels of depression and to what extent that should be considered in making healthcare recommendations to support very young children with SCD.

Routine depression screening during pediatric SCD visits could be feasible using brief tools like the Patient-Reported Outcomes Measurement Information System—Emotional Distress—Depression—Short Form (PROMIS—Depression).^18^ Regular screening would help identify changes in caregiver mental health as children age and disease complications potentially increase. For caregivers showing elevated symptoms, clinics could develop referral pathways to mental health services or family-centered programs that support both caregiver well-being and disease management skills (eg, Part C early intervention services). For caregivers with low depression scores, preventive approaches such as peer support groups or care coordination might help maintain psychological health while building coping strategies before challenges intensify. Yet, depression screening among caregivers remains limited.

We screened for depression among caregivers of children 0-5 years of age with SCD using the PROMIS—Depression.^18^ The PROMIS tool is short, with only 8 items that can be automatically scored, and allows for quick screening within clinical visits. Given mixed findings in the literature and the unique challenges facing this population, this study aimed to (1) assess depression levels among caregivers of children 0-5 years with SCD using the PROMIS Depression screening tool and (2) examine the association between neighborhood disadvantage using the Area Deprivation Index and caregiver depression.

## MATERIALS AND METHODS

The Institutional Review Boards at Washington University School of Medicine and St. Jude Children’s Research Hospital approved this cross-sectional cohort study as part of a larger qualitative study.^19^^,^^20^ Caregivers of children 0-5 years of age with SCD were approached by phone or following clinic visits using a convenience sampling approach. Caregivers were included if they were the primary caregiver for a child <5 years with SCD and spoke English. Caregivers were excluded if their child had additional diagnoses that could affect development (eg, Autism Spectrum Disorder). Following consent, caregivers were asked to provide demographic information and complete the PROMIS Depression screening tool during clinic visits or scheduled follow-up calls.

The PROMIS depression scale includes 8 Likert-scale questions, ranging from 1 (never) to 5 (always), and has strong psychometric properties.^21^^,^^22^ T-scores range from 0 to 100, where a score of 50 is the mean of the general population (1 SD = 10).^23^ Scores ≥60 are typically considered indicative of moderate depression, whereas scores >70 are considered severe. Relative environmental risk was determined using the ADI. The ADI is a validated metric of neighborhood disadvantage utilizing education, employment, housing, and poverty measures to create a composite score.^24^ ADI scores range from 1 to 100, with higher scores indicating greater environmental risk. For descriptive purposes, ADI scores were categorized as high deprivation (71-100), moderate deprivation (50-70), and low deprivation (<50).^25^ For statistical analysis examining the relationship with depression, continuous ADI scores were used. Analyses were completed using R statistical software (version 4.4.8, 2025). Descriptive statistics were calculated to characterize demographic information and the distribution of scores. The relationship between ADI and PROMIS Depression scores was examined using the Pearson correlation coefficient. A significance threshold of *P* < .05 was used to determine statistical significance.

## RESULTS

Among 127 caregivers identified as eligible, 47 (37%) participated. More details on recruitment and demographic information are published elsewhere.^20^ We did not collect demographic information from those who declined participation, limiting our ability to assess systematic differences between participants and non-participants. Given that 46 of 47 participants were mothers, findings primarily reflect maternal caregivers’ experiences, though we retained the single father participant to maintain our complete cohort.

**Figure 1.**
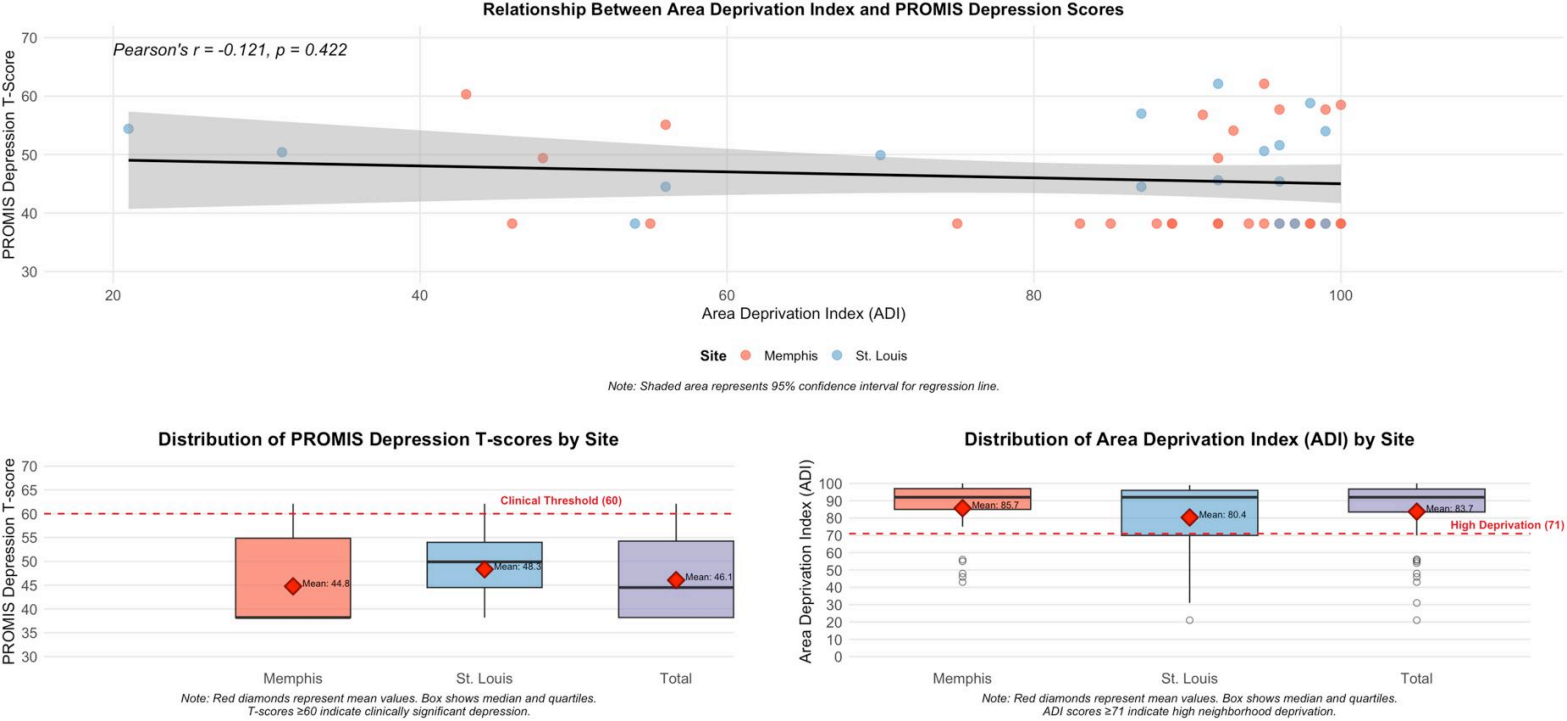
Relationship between area deprivation index and PROMIS depression scores.

The mean PROMIS Depression T-score for the cohort was 46.05 (SD = 8.66). Most caregivers endorsed responses on the PROMIS Depression screening tool similar to the general population (*n* = 44, 94%), with 3 endorsing responses indicative of elevated depression of 1 SD above the mean (T Score > 60). The large majority of participants resided in areas of high neighborhood disadvantage (77%, ADI 71-100), with some in moderate (11%, ADI 50-70) and some in low (11%, ADI <50) deprivation areas (Figure 1).^26^ As seen in Figure 1, which displays both distributions and mean values, average PROMIS scores were similar across sites (Midsouth mean = 44.76, SD = 9.08; Midwest mean = 48.33, SD = 7.59). Statistical analysis revealed no significant association between neighborhood disadvantage (ADI) and caregiver responses on the PROMIS Depression screening tool (*P *= .42).

## DISCUSSION

Caregivers in this study responded similarly to the general population on a brief depression screening tool, which suggests a potential intervention window that has been underexplored in SCD management. While some research has documented the substantial psychological burden experienced by caregivers of older children with SCD,^27–29^ our findings replicate other reports suggesting low rates of depression among caregivers of young children with SCD,^8^ and indicate that families with very young children may represent an optimal time period for preventive interventions.

The period spanning the first years of life presents a unique opportunity for comprehensive family-centered interventions. Caregivers may have greater capacity to participate in education interventions during this period of early development, before starting school, and when disease complications are often less intense. Early intervention programs (occupational, physical, and speech therapy for children 0-3 years) are valued by caregivers and providers,^20^^,^^30^ have demonstrated feasibility,^31^ and could address multiple domains simultaneously: optimizing the home environment for developmental support, building caregiver capacity for disease management, and establishing robust support systems before crisis situations arise. Despite the potential benefit of early intervention to remediate developmental delay and support caregiver involvement in managing this chronic condition, children with SCD are overlooked for federally funded, home-based early intervention.^32^

Intervention during this period of rapid growth and development could offer families the strongest opportunity to develop kindergarten readiness among children with SCD. There was no association between neighborhood disadvantages and depression, which suggests that neighborhood factors alone may not be related to caregiver psychological functioning. This indicates that caregivers, regardless of financial environment, could potentially engage in early intervention. Among this cohort, environmental risk factors characterized by the ADI were not associated with elevated depressive symptoms, suggesting that protective factors or coping mechanisms might be present, though longitudinal research is needed to understand these relationships and how they relate to participation in preventive interventions.

This study has several limitations that should be considered. Our cohort consisted almost entirely of mothers (46/47), limiting generalizability to fathers, grandparents, and other family caregivers. This composition reflects prior studies that have worked with primary caregivers of young children with SCD. Additionally, this study was not designed to detect between-group differences. The cross-sectional design limits generalization in understanding depression scores across ages and changes in disease severity and suggests that future work should explore the validity of brief screening tools to assess caregiver mental health over time. Finally, while the PROMIS tool enabled quick screening feasible within clinical visits, it captures only depressive symptoms rather than the full spectrum of caregiver burden, including stress, anxiety, or practical challenges families face.

## CONCLUSION

In this cohort of 47 caregivers of young children with SCD, depressive symptoms were within normative expectations, with 94% reporting scores similar to the general population. These findings suggest caregivers of young children with SCD may be well-positioned to engage in preventive interventions during this early developmental period. Future research should examine whether low rates of depression persist over time and determine the desire for, and impact of education-focused interventions to support caregivers of children with SCD during the first years of life.
